# The Story of the Insane from Year to Year

**Published:** 1890-11-01

**Authors:** 


### THE STORY OF THE INSANE FROM YEAR TO YEAR.

[Annual Reports, Pamphlets, &c., should be addressed to THE EDITOR The Lodge, Porchester Square, London, W.]

### DISTRICT ASYLUM FOR ARGYLL AND BUTE.

During the year 1889 there was a decrease of three patients on the numbers of the year previous—a very unusual state of things. There were 84 admissions; 66 were discharged, and 21 died. Thrown into percentages, these numbers show that the recoveries stand at 49 per cent., and the deaths at 6 per cent.; the former being high and the latter low. The committee of visitors "express their perfect satisfaction with the management of the asylum." Dr. Cameron's report is clear and concise. It seems that measles broke out in the asylum, and seven patients and five attendants caught it. The disease would seem to have been of a rather severe type, as there were two deaths. Having no hospital for infectious diseases, the tailors' shop was converted into a temporary one. Why will so many asylums go on year after year without making the necessary provision for the treatment of fevers?

### PERTH DISTRICT ASYLUM.

Here we find no report from the committee of visitors; but the usual report of the medical superintendent is given, as well as three reports from the Commissioners in Lunacy. The asylum, like so many of the Scotch district asylums, is a small one, containing only 318 patients. The percentage of recoveries was low, being 17·9 on the admissions, and the death-rate was 7·5 on the average number resident. Among the deaths was one from enteric fever. The patients had been some time in the asylum, and the "attack was not traceable to any defect in the sanitary arrangements of the building; and, as no other resident showed the slightest symptom of the disorder, this may be regarded as a sporadic, isolated, or spontaneous case." So says Dr. Campbell, and we think the details of such cases should have a wider circulation than an asylum report can give. If enteric fever can originate spontaneously, why not other fever or cholera? The Commissioners say, "the asylum was everywhere in excellent order, and the condition of the inmates was in all respects satisfactory."

### KENT COUNTY ASYLUM, CANTERBURY.

The patients in this asylum increased by 39 between January 1, 1889, and the last day of December of the same year, there being resident on that day 795 cases. The admissions rose to 175, being the highest recorded number since the year 1883. Fifty-eight patients were discharged recovered, showing a percentage on the admissions of 33. There were 69 deaths, and eight of these were owing to phthisis. The Committee of Visitors do not publish their report.

The Commissioners in Lunacy suggest improvements in various directions; for instance, in the infirmary wards. They also recommend that more tell-tale clocks be supplied, and that the wards should be better lighted.

### IPSWICH BOROUGH ASYLUM.

Like most borough asylums, the number of patients resident here is really too few for proper classification and organization. At the close of 1889 only 259 patients were in the asylum; 111 patients were admitted; 34 were discharged; and 25 died. The recoveries calculated on the admissions stand at 19 per cent., and the deaths at 11 per cent. on the average number resident. Mr. Rowe deplores the fact that so few marks of recognition from the outside public are made to patients in asylums. It is doubtful, however, whether his appeal will have any effect. The benevolent pass by the asylum. The visitors refer to the death of their late superintendent, Dr. Chevallier, and they mention that Mr. Rowe was unanimously appointed his successor.

